# Ar^+^ implantation-induced tailoring of RF-sputtered ZnO films: structural, morphological, and optical properties

**DOI:** 10.3762/bjnano.16.66

**Published:** 2025-06-11

**Authors:** Manu Bura, Divya Gupta, Arun Kumar, Sanjeev Aggarwal

**Affiliations:** 1 Department of Physics, Kurukshetra University, Kurukshetra, 136119, India

**Keywords:** AFM, diffuse reflectance, GXRD, polycrystalline, ZnO films

## Abstract

Radio frequency-sputtered zinc oxide films are implanted with 30 keV Ar^+^ ions at various fluences ranging from 1 × 10^15^ to 2 × 10^16^ ions·cm^−2^. Raman spectra reveal the presence of the E_2_ (low), E_2_ (high), and A_1_ (LO) Raman modes in pristine and implanted ZnO films. A gradual fall and rise in peak intensity of, respectively, the E_2_ (high) and A_1_ (LO) Raman modes is observed with increases in ion fluence. However, the E_2_ (low) mode broadens and merges completely with disorder-induced broad band at higher fluences. Moreover, the deconvolution of the A_1_ (LO) Raman peak affirms the presence of defect-related Raman modes in the implanted samples. A gradual reduction in crystallinity of the implanted ZnO films with increasing ion fluence is observed in grazing incidence angle X-ray diffraction patterns. Atomic force microscopy images show grain size reduction and a fall in the surface roughness value of films after implantation. The implantation-induced structural modifications are further correlated with the variation in diffuse reflectance, Urbach energy, and optical bandgap. The low reflectance values of implanted films assure their suitability as transparent windows and anti-reflective coating in various optoelectronic devices.

## Introduction

Zinc oxide has emerged as a promising material for device fabrication in different fields, namely, spintronics, nanoelectronics, and photonics [1–2]. It possesses a wide bandgap of 3.37 eV [3] and has a large exciton binding energy of about 60 meV [4], which assures the stability of ZnO film-based devices such as liquid crystal displays [5], solar cells [6], and light-emitting diodes [7]. There are numerous methods for synthesizing ZnO films, including pulsed laser deposition, spray pyrolysis, radio frequency (RF) sputtering, and sol–gel techniques. Here RF sputtering is preferred over other methods because it provides high deposition rates and uniform growth of films with good reproducibility [4]. The physical properties of grown ZnO films can be tuned by altering various growth parameters and employing post-deposition treatments such as ion implantation and thermal annealing.

Ion implantation has proven a versatile tool to control material properties by inducing damage and introducing defects in the host matrix in a controlled manner [8]. It offers the advantage of controlling the amount of energy transferred to the host system by selecting the desired ion energy, mass, and fluence [9]. Different types of lattice vacancies, defects, and interstitials are induced through the interaction between energetic ions and the host material, resulting in structural modification and thus alteration in lattice dynamics of the host material [10].

The implantation-induced disorder can be qualitatively examined using Raman spectroscopy, which is a well-established and non-destructive method to determine crystal structure, lattice defects, and dynamics. Since ZnO is a polar semiconductor, the phonon–electron interaction produces longitudinal optical (LO) phonon modes, whose long-range behavior considerably affects the efficacy of optoelectronic devices [11]. Thus, a detailed study of the evolution of phonon modes is needed to utilize implanted ZnO films effectively in such devices. The activation of Raman modes in implanted films depends on various implantation parameters, namely, ion energy, mass, and fluence.

The origin of these optical phonon modes is ascribed to the formation of oxygen vacancies, which are supposed to be electron carriers in ZnO. Therefore, the evolution of the A_1_ (LO) mode acts as indirect evidence of a rise in carrier concentration, which can in turn alter the optical bandgap. Moreover, the presence of foreign ions in the ZnO film lattice can create an impact on its surface roughness and particle size.

Previous reports available discuss the implantation-induced optical longitudinal phonon symmetry in ZnO films using heavy ions with high energy and low implantation fluences [12–15]. Singh et al. [12] observed the evolution of symmetry-forbidden and A_1_ (LO) modes in 120 MeV Au^9+^ ion-irradiated ZnO films. Ying et al. [13] described an A_1_ (LO) mode in the Raman spectra of energy-dependent and dose-dependent krypton ion-implanted ZnO film after varying the fluence in the range from 5 × 10^13^ to 2.5 × 10^15^ ions·cm^−2^. Gupta et al. [14] have investigated the activation of the A_1_ (LO) mode and the production of a broad band at the lower Raman shift side in ZnO films implanted with 300 keV argon and 1.2 MeV xenon ions with varying fluence from 1 × 10^14^ to 3 × 10^15^ ions·cm^−2^. Gautam et al. [15] reported the presence of an A_1_ (LO) Raman mode and a disorder-induced band at low wavenumbers in cadmium-doped zinc oxide films irradiated using 120 MeV Ag^9+^ and 80 MeV O^6+^ ions at fluences of 1 × 10^13^ and 3 × 10^13^ ions·cm^−2^.

Further, few studies [16–17] reported the emergence of optical longitudinal phonon symmetry in ZnO films implanted at lower ion beam energies. Zhiguang et al. [16] have observed the appearance of a longitudinal phonon mode in 80 keV nitrogen ion-implanted ZnO films at different fluences. Kennedy et al. [17] have reported enhancement in the disordered phase and an A_1_ (LO) mode in 23 keV co-implanted (H^+^ and N^+^ ions) ZnO films. But in these two above-quoted reports nitrogen ions were used for implantation. Nitrogen ions act as n-type doping and can alter the stoichiometry of ZnO films, which is not desirable in certain optoelectronic devices [10–11]. Hence, we have used Ar^+^ ions for implantation, which produce less lattice distortions than nitrogen ions. This is because argon ions are heavier and larger than nitrogen ions.

Also, in the above-quoted studies the authors did not study the effect of the evolution of longitudinal optical A_1_ (LO) and symmetry-disallowed Raman modes on the surface morphological and optical characteristics (Urbach energy and optical bandgap). In fact, in the existing literature, there are barely any studies that have addressed the impact of the evolution of A_1_ (LO) modes on surface morphology and optical properties in low-energy regimes, although the variation in surface parameters and optical characteristics can significantly impact the applicability of ZnO films in semiconductors, spintronics, solar cells, and green energy industries [3,18].

This motivated us to investigate the emergence of Raman longitudinal optical modes and their correlation with morphological and optical properties using low-energy Ar^+^ beams in ZnO films. Here, argon ions were chosen because of the inert nature, which means that any changes in properties of the implanted ZnO films are attributed solely to implantation-induced effects.

In the present study, ZnO films were implanted with 30 keV Ar^+^ at fluences varying from 1 × 10^15^ to 2 × 10^16^ ions·cm^−2^. Surface variables (roughness and particle size), structural variables (crystallite size and dislocation density), and optical properties (diffuse reflectance, Urbach energy, and optical bandgap) were studied in response to a rise in ion fluence. The ion implantation-induced lattice disorder and lattice damage as functions of the ion fluence were studied in terms of displacement produced per atom in the host lattice calculated using TRIM simulations [19] and were correlated with changes in Urbach energy. The films are versatile in developing high-performance electro-optical and spintronic devices [18].

## Experimental

**Figure 1:**
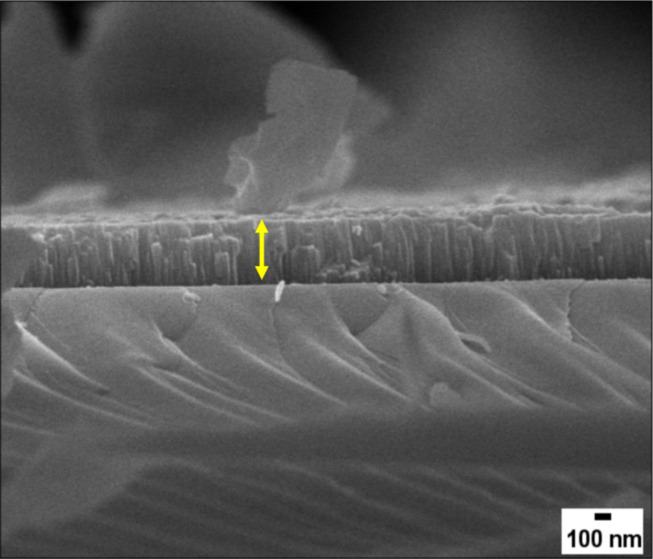
Cross-sectional FESEM images of pristine ZnO film. Here yellow arrow represents the thickness of grown film.

ZnO films are grown on a quartz substrate (1 × 1 cm^2^) using a ZnO (99.99%) target (2″ diameter and 3 mm thickness) in a radio frequency (RF) sputtering system. The quartz substrate is ultrasonically cleaned using acetone and, finally, isopropyl alcohol before the experiment. The sputtering chamber is pumped to a base pressure of 1.2 *×* 10^−6^ Torr; then a mixture of nitrogen and argon gas is introduced into the sputtering chamber with flows of 1.8 and 10.0 sccm, respectively. When the pressure inside the chamber has stabilized, the sputtering power is set to a value of 80 W. The sputtering is performed at a pressure of 1.8 × 10^−5^ Torr at room temperature with a deposition rate of 0.4–0.5 Å·s^−1^. A spectroscopic ellipsometer is used to calculate the thickness of the pristine ZnO films. An appropriate physical model is designed and fitted using different ellipsometry parameters to obtain the least root mean square error. The thickness of the as-grown ZnO films was found to be around 296 ± 6 nm. Moreover, the thickness of ZnO films calculated using cross-sectional FESEM images was of the same order as the thickness calculated from spectroscopic ellipsometry (Figure 1).

After deposition, films are implanted with 30 keV Ar^+^ ions at different fluences of 1 × 10^15^, 5 × 10^15^, 1 × 10^16^, and 2 × 10^16^ Ar^+^ cm^−2^ using the 200 kV ion accelerator facility at Ion Beam Centre, Kurukshetra University. The implantation is carried out at normal incidence for all fluences. The electronic energy loss of 30 keV Ar^+^ ions in ZnO films is 18.73 eV·Å^−1^, while the nuclear energy loss is 9.610 × 10^3^ eV·Å^−1^, calculated using SRIM-2008 [19]. The projected range of 30 keV Ar^+^ ions in the ZnO lattice is 25.9 ± 13.7 nm.

The crystalline structure is studied using a Bruker AXS D8 Advance X-ray diffractometer operating in grazing incidence geometry using Cu Kα radiation (λ = 1.5406 Å). The scans are obtained at an incidence angle of 0.5°. The Raman spectra of ZnO films before and after implantation are recorded at room temperature using a WITec alpha300 RA Raman spectrometer under excitation with a 532 nm solid-state diode laser operated at 10 mW. The topography of the films is examined using atomic force microscopy (AFM) with a Bruker Multimode 8 instrument. The surface morphology of pristine and implanted films is further studied using field-emission scanning electron microscopy (FESEM) along with energy dispersive X-ray spectroscopy (EDS). Cross-sectional images are also obtained to evaluate the thickness of ZnO film. The optical properties of pristine and implanted ZnO films are investigated using a Shimadzu UV–visible–NIR spectrophotometer (UV-3600Plus) employed with Integrating Sphere Assembly (ISR-603) in the wavelength range of 200–800 nm.

## Results and Discussion

### Structural analysis

#### Grazing incidence X-ray diffraction

**Figure 2:**
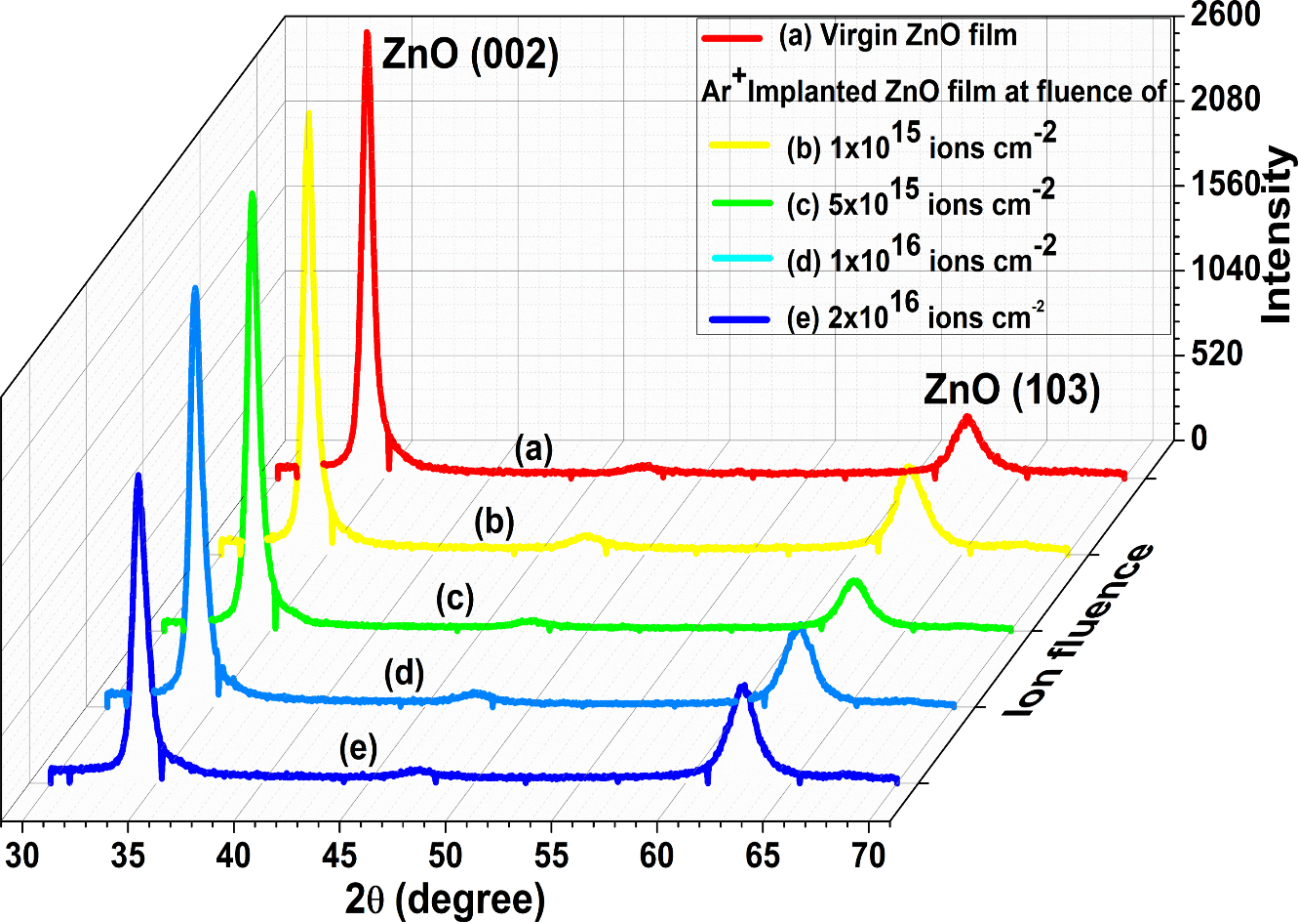
GXRD pattern of pristine and Ar^+^-implanted ZnO films at various fluences.

The grazing incidence X-ray diffraction (GXRD) patterns of pristine ZnO and argon-implanted ZnO films at various ion fluences are depicted in Figure 2. The coexistence of two diffraction peaks (Figure 2) depicts the polycrystalline nature of films. The diffraction peaks centered at 2θ values of 34.23° and 62.59° corresponding to (002) and (103) planes, respectively, confirm the wurtzite structure (JCPDS No. 36-1451) of pristine samples (Figure 2a) [20]. The intense peak centered at 2θ = 34.23° indicates the growth of samples along the *c* axis, that is, in the [002] direction, which has the lowest surface energy. The existence of a peak related to the (103) planes can be attributed to the presence of intrinsic defects in the films [21–22]. The presence of the same diffraction peaks in the GXRD pattern of implanted samples (Figure 2b–e) suggests the occurrence of identical crystal structures after implantation.

To study the effect of implanted ions on the structure of the films, the more intense (002) peak is further analyzed. The intensity of the peak reduces with increasing ion fluence, revealing a reduction in crystallinity. This is due to argon ion implantation-induced lattice damage. Yet, even at the highest fluence, complete amorphization is not detected.

**Figure 3:**
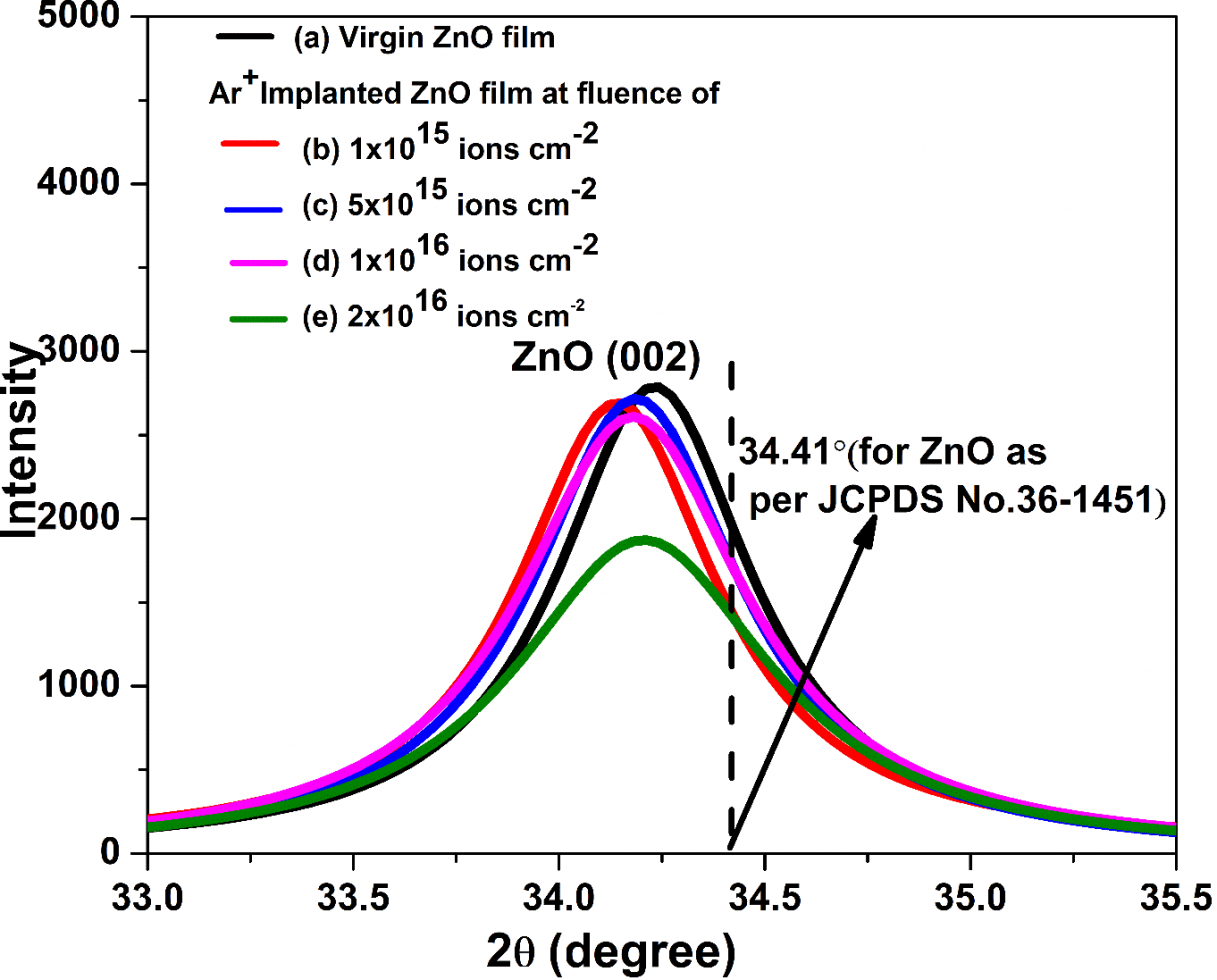
Shift in peak position of the (002) peak centered at 34.23° for all samples. 34.41° is the Bragg angle of the (002) reflection as per JCPDS No. 36-1451.

ZnO films were implanted with 30 keV Ar^+^ ions. The energy used here was a low energy; also, argon is lighter than zinc. Because of this, the irradiation did not cause a significant shift in peak positions with increasing ion fluence, but it is observable. The shift in peak position and the variation in peak intensity of the (002) peak at 34.41° with increase in ion fluence is given in Figure 3 and Table 1.

**Table 1:** Variation in peak position, intensity, and shift in peak position of the (002) peak centered at 34.23° with increase in ion fluence.

Ion fluence (ions·cm^−2^)	2θ (°) from literature	2θ (°) in present work	Peak intensity	Shift in peak position (°)

pristine	34.41	34.23	2781	−0.18
1 × 10^15^	–	34.14	2654	−0.27
5 × 10^15^	–	34.18	2735	−0.23
1 × 10^16^	–	34.18	2612	−0.23
2 × 10^16^	–	34.20	1887	−0.21

For more detailed information regarding the structural evolution of implanted ZnO films, the crystallite size (*D*), microstrain (ε), and dislocation density (δ) values are calculated from the (002) peaks using the following equations [23]:


[1] $$D=\frac{0.9\lambda }{\beta \cos\theta },$$

[2] $$\varepsilon =\frac{\beta }{4\tan\theta },$$

[3] $$\delta =\frac{1}{D^{2}}.$$

In the above relations, λ is the wavelength of the incident Cu Kα radiation (1.5406 Å), β represents the full-width at half maximum (FWHM), and θ is the peak position. The variation in values of these parameters is shown in Table 2.

**Table 2:** Variation in FWHM, crystallite size *D*, dislocation density δ, and microstrain ε of pristine and Ar^+^-implanted ZnO films at various fluences.

Ion fluence (ions·cm^−2^)	2θ (°)	FWHM (°)	Crystallite size (*D*) (nm)	Dislocation density (δ) (10^16^ m^−2^)	Microstrain (ε) (10^−3^)

pristine	34.23	0.577	14.42 ± 0.35	0.48	8.16
1 × 10^15^	34.14	0.588	14.16 ± 0.47	0.49	8.33
5 × 10^15^	34.18	0.618	13.46 ± 0.44	0.55	8.75
1 × 10^16^	34.18	0.669	12.45 ± 0.45	0.64	9.47
2 × 10^16^	34.20	0.759	10.97 ± 0.47	0.83	10.73

The crystallite size of the pristine sample is found to be 14.42 ± 0.35 nm. It decreases slowly with the rise in implantation fluence and achieves a value of 10.97 ± 0.47 nm at the highest ion fluence due to a reduced crystallinity of the implanted films. Moreover, argon atoms can reside on substitutional sites of the ZnO lattice, which causes strain in the implanted layers; thus, the microstrain values increase with fluence [24]. Strain in implanted ZnO films arises primarily from lattice mismatch, which is due to the difference in thermal expansion coefficients between film and substrate. Also, when argon ions are implanted into the ZnO lattice, they create defects and dislocations. This creates lattice strain, which increases with ion fluence. The size and type of the implanted ions, as well as the dose, can affect the amount of strain introduced [24]. It is observed that dislocation density values increase with the rise in implantation fluence, which can be attributed to the fact that an enormous amount of energy is transferred to the lattice when the ion beam travels through the sample quickly, which generates dislocations.

#### Raman spectroscopy

**Figure 4:**
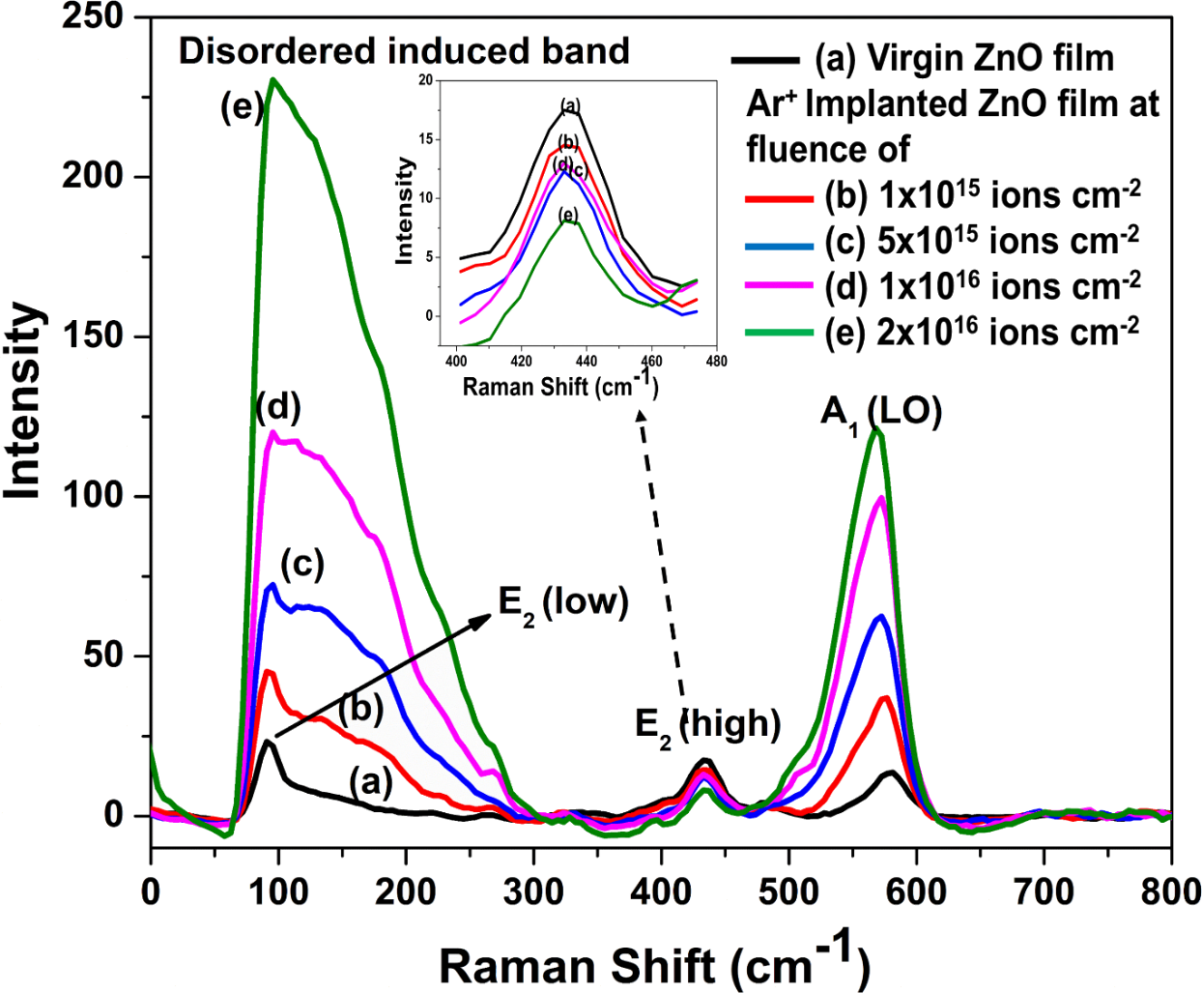
Raman spectra of (a) pristine and Ar^+^-implanted ZnO films at various fluences of (b) 1 × 10^15^, (c) 5 × 10^15^, (d) 1 × 10^16^, and (e) 2 × 10^16^ ions·cm^−2^ with inset representing the peak related to the E_2_ (high) mode.

Figure 4 reveals the Raman spectra of pristine and 30 keV argon-implanted ZnO films at various fluences. The spectrum of the pristine film (Figure 4a) show peaks at 96, 433, and 577 cm^−1^, which correspond to E_2_ (low), E_2_ (high), and A_1_ (LO) modes of ZnO respectively. The prominent peaks corresponding to E_2_ (low) and E_2_ (high) are characteristic peaks related to the wurtzite crystal structure of ZnO, which points toward the good crystallinity of our films.

Moreover, the presence of the A_1_ (LO) and E_2_ (high) modes indicates the growth of the film along the *c* axis, which is also confirmed using GXRD. The intensity of the peak related to the A_1_ (LO) mode is relatively weak in the pristine film (Figure 4a). The A_1_ (LO) mode evolves because of defects present in the film in the form of oxygen vacancies, zinc interstitials, and their complexes. For the case of ZnO films implanted at 1 × 10^15^ ions·cm^−2^ fluence (Figure 4b), the intensity of the E_2_ (low) and A_1_ (LO) modes increases, while the peak intensity of the E_2_ (high) modes decreases. Besides this, a broad band started to appear around 104 to 200 cm^−1^, which is assigned as a disorder-induced band due to lattice disorder induced by ion implantation [14]. With further increase in fluence to 5 × 10^15^ ions·cm^−2^ (Figure 4c), phonon modes corresponding to A_1_ (LO) symmetry intensify, and those corresponding to E_2_ (high) weaken. Also, the peak related to the E_2_ (low) mode starts to merge with the disorder-induced broad band. At 1 × 10^16^ ions·cm^−2^ fluence (Figure 4d), the peak related to the E_2_ (low) mode merges completely with the disorder-induced broad band, while the phonon mode corresponding to A_1_ (LO) symmetry intensifies and the intensity of the phonon mode referred to as E_2_ (high) decreases. Last, at the highest fluence (Figure 4e), the intensity of disorder-induced broad band surpasses the phonon mode related to A_1_ (LO) symmetry, and the peak intensity related to the E_2_ (high) phonon mode diminishes. The decrease in intensity of the E_2_ (high) phonon peak can be attributed to the evolution of defects in the oxygen (O^2−^) sublattice due to energy deposition via ion implantation. This is also correlated with enhancement in the intensity of the disorder-induced band and a decrease in crystallinity along the *c* axis as depicted by GXRD.

Furthermore, for a better understanding of the evolution of defects with implantation, position and FWHM of the peak corresponding to the A_1_ (LO) phonon mode of all samples is displayed in Table 3. It is observed that for all the samples, the A_1_ (LO) phonon mode exhibits softening and broadening with the rise in argon ion fluence from 1 × 10^15^ to 2 × 10^16^ ions·cm^−2^. It is well known that a shift of the peak position of phonon modes occurs because of strain present in the film. The broadening of peaks occurs because of the fast decay of phonons or an anharmonic process due to damage [25]. One can determine the phonon lifetime from the Raman spectra using the energy–time uncertainty equation [25]:


[4] ΔE⋅τ=h2π

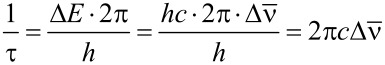


Here 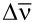 represents Raman shift, which is of the order of the FWHM (Γ) of the Raman mode; thus, the lifetime is determined employing the following relation [25]:


[5] $$\frac{1}{\tau }=2\pi c\Gamma .$$

The lifetime related to the phonon is calculated using Equation 5 and summarized in Table 3; the values are of the order of picoseconds and match well with the literature [26–27]. It is found that the lifetime of the A_1_ (LO) mode is becoming shorter with the rise in argon ion fluence, which can be correlated with the emergence of the defect-induced band. Moreover, phonon softening relates to tensile stress, while phonon stiffening relates to compressive stress. Thus, all argon ion-implanted ZnO films show phonon softening, which indicates that tensile stress is produced in the films with an increase in argon ion fluence. This can be ascribed to expansion in volume due to implanted ions since argon ions are inert in nature, which prevents them from reacting with host ions. This leads to the accumulation of inert ions at the interstitial sites of ZnO, which produces stress in the material [14].

**Table 3:** Variation in the position of peak and FWHM corresponding to the A_1_ (LO) mode, phonon lifetime, and number of displacements produced per atom (dpa) as a function of ion fluence.

Ion fluence (ions·cm^−2^)	Peak position (cm^−1^) of A_1_ (LO) mode	FWHM (cm^−1^) of A_1_ (LO) mode	Lifetime (τ) picoseconds	dpa

pristine	577	38.2	0.138	–
1 × 10^15^	569	46.6	0.113	0.45
5 × 10^15^	565	49.3	0.107	2.25
1 × 10^16^	564	49.8	0.106	4.51
2 × 10^16^	562	50.9	0.104	9.03

Additionally, it is observed that the intensity of the disorder-induced band rises with the rise in Ar^+^ fluence. This is attributed to the fact that ion implantation produces lattice disorder or lattice damage, which is studied in terms of the fluence of implanted ions and displacements produced per atom (dpa) in the host matrix through implantation [28]. The value of dpa can be calculated via TRIM simulations using the following relation [14]:


[6]

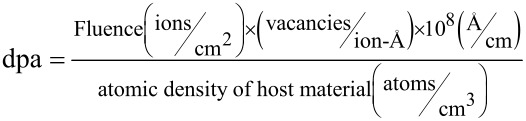



**Figure 5:**
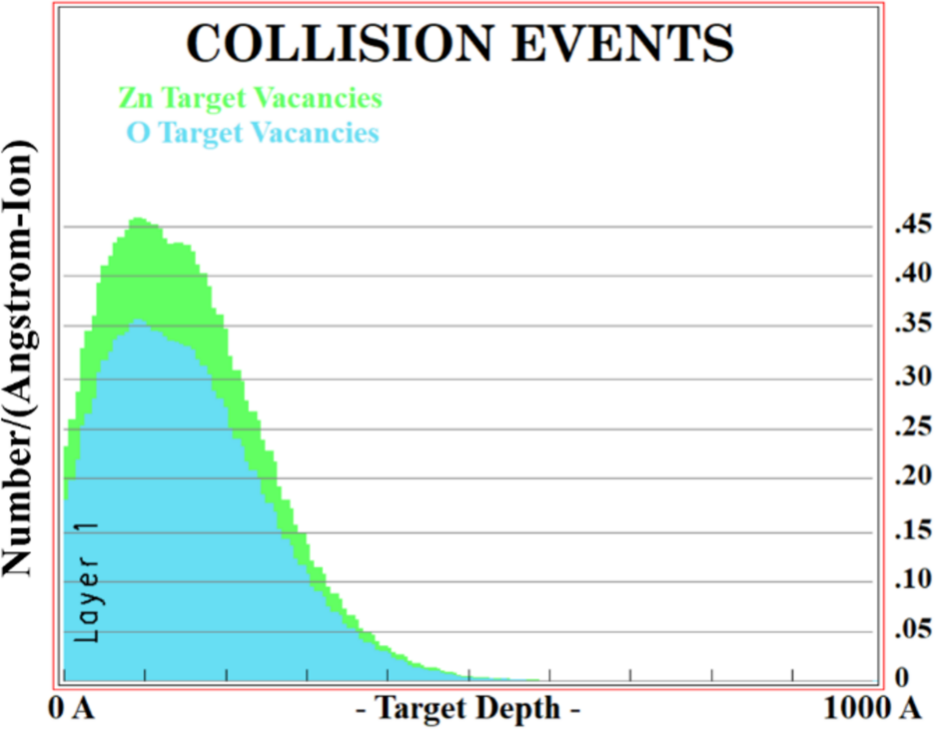
Number of zinc and oxygen vacancies created by the argon ion beam in the ZnO target calculated using TRIM simulations.

The above equation depicts the number of vacancies created per ion per angstrom, which can be calculated from TRIM simulations as shown in Figure 5. For ZnO, the atomic density value is 8.30 × 10^22^ atoms/cm^3^, and the displacement energy for both zinc and oxygen is 56 eV.

It is to be noticed from Table 3 that dpa increases with increases in argon ion fluence. Thus, the rise in intensity of the disorder-induced band can be ascribed to an increase in dpa, which leads to lattice disorder [14].

**Figure 6:**
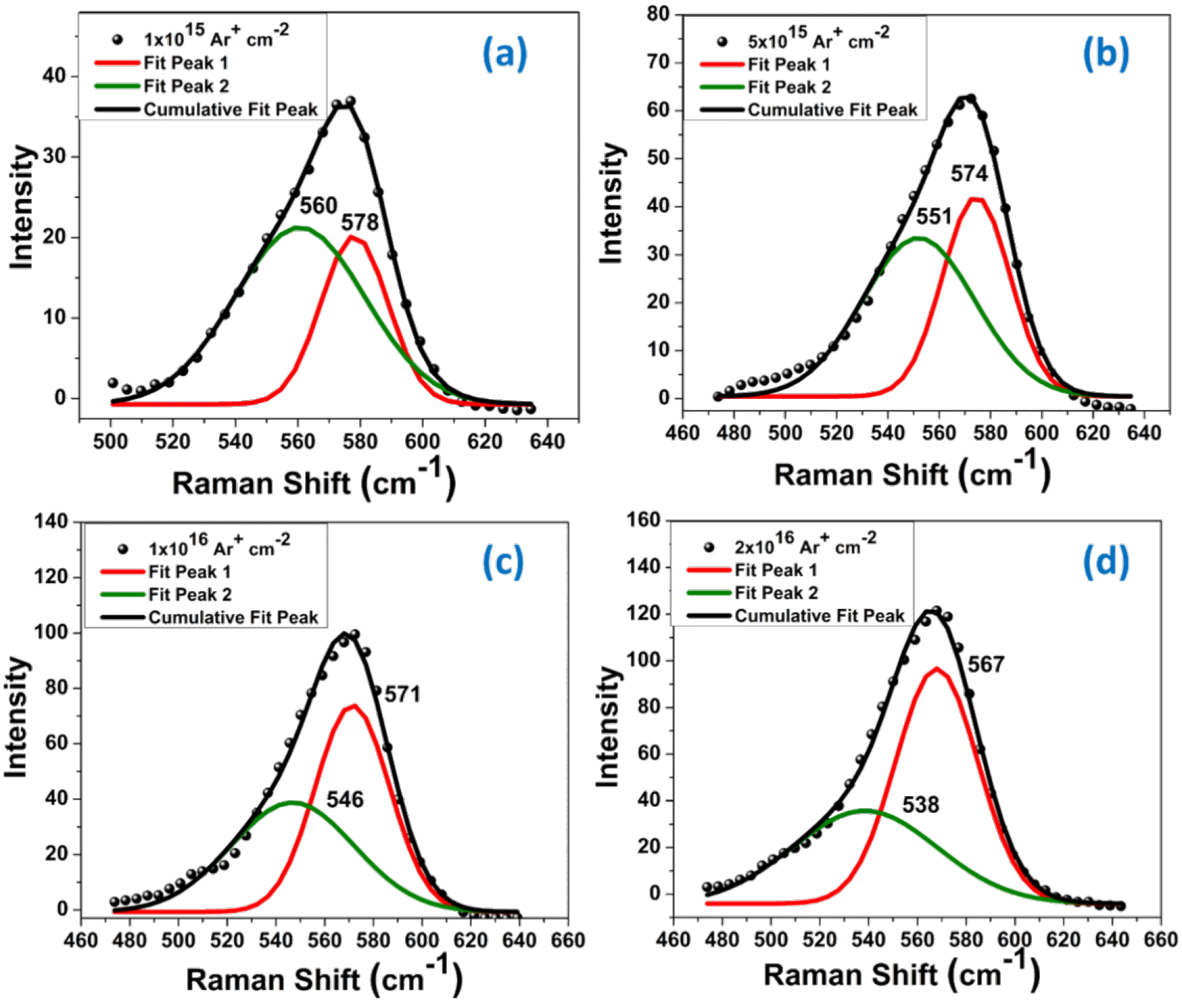
Deconvolution of the A_1_ (LO) Raman peak of ZnO films implanted at various fluences of (a) 1 × 10^15^, (b) 5 × 10^15^, (c) 1 × 10^16^, and (d) 2 × 10^16^ ions·cm^−2^.

The peak corresponding to the A_1_ (LO) mode of implanted samples is deconvoluted as shown in Figure 6. This type of scattering from the K–M point of the Brillouin zone is symmetrically forbidden. Gupta et al. [14] have also reported such behavior of the A_1_ (LO) Raman mode in 300 keV argon ion-implanted ZnO films. Mondal et al. [29] and Li et al. [30] have ascribed these peaks centered at 577 and 554 cm^−1^ to oxygen vacancies and zinc interstitials, respectively. Moreover, the peak related to the A_1_ (LO) mode both at the Γ and K–M points of the Brillouin zone shows softening and broadening with increasing argon ion fluence. Also, enhancement in peak intensity of both the peaks reveals the increases in lattice defects with increasing ion fluence.

### Morphological analysis

#### Atomic force microscopy

**Figure 7:**
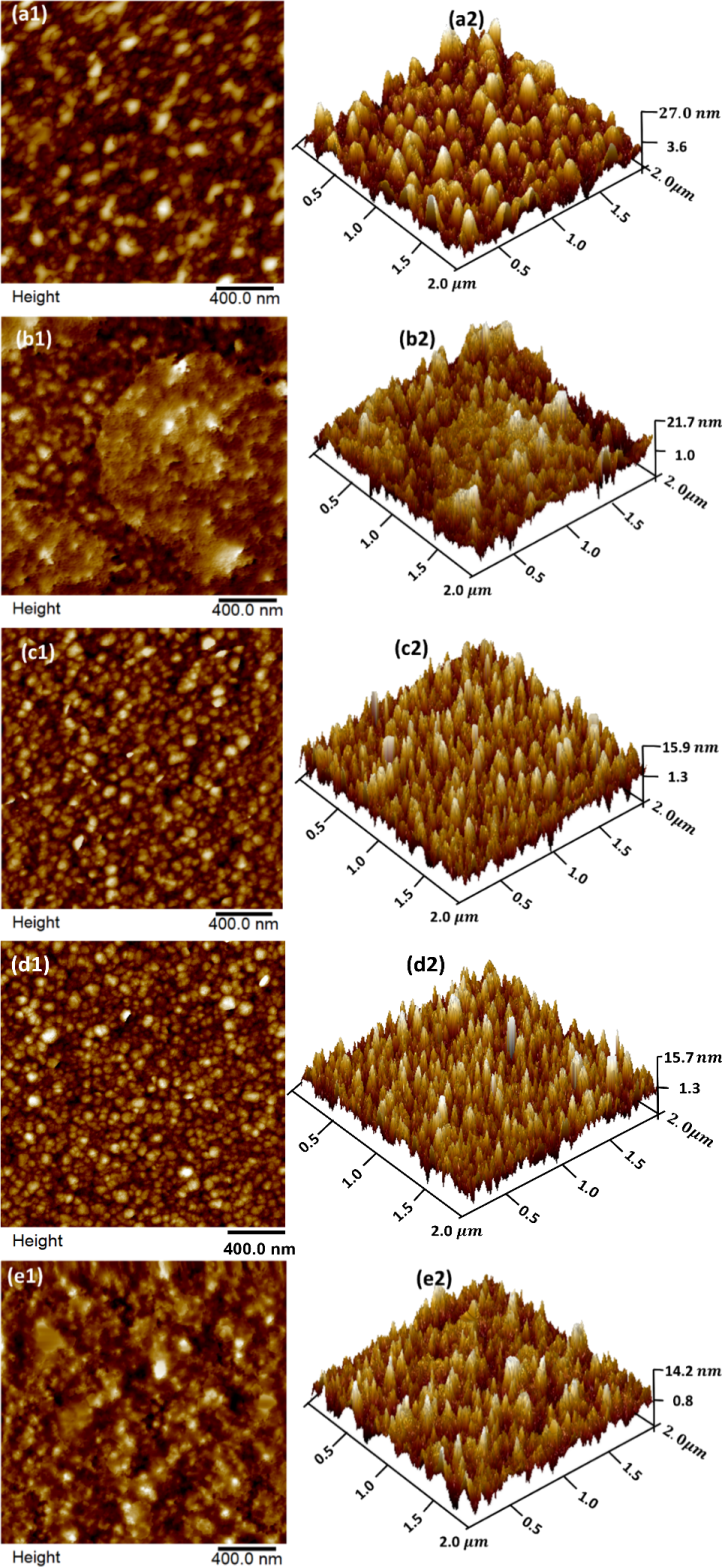
2D and 3D AFM images of pristine (a1, a2) and Ar^+^-implanted ZnO films at fluences 1 × 10^15^ (b1, b2), 5 × 10^15^ (c1, c2), 1 × 10^16^ (d1, d2), and 2 × 10^16^ ions·cm^−2^ (e1, e2).

The surface morphology of pristine and 30 keV Ar^+^ ion-implanted ZnO films is studied using AFM. Figure 7 represents 2D and 3D AFM images at the scale 2 µm × 2 µm of the pristine film (Figure 7a) and films implanted at four different fluences, viz. 1 × 10^15^ (Figure 7b), 5 × 10^15^ (Figure 7c), 1 × 10^16^ (Figure 7d), and 2 × 10^16^ ions cm^−2^ (Figure 7e).

All the images have been analyzed using Nanoscope analysis software provided with the AFM to determine the particle size and surface root mean square (RMS) roughness values for different implanted samples. The results are shown in Table 4.

**Table 4:** Variations of particle size and surface RMS roughness values of pristine and Ar^+^-implanted ZnO films as functions of ion fluence.

Fluence (ions·cm^−2^)	Particle size (nm)	RMS roughness (nm)

pristine	74.41 ± 0.71	6.92 ± 0.22
1 × 10^15^	63.00 ± 0.25	5.88 ± 0.67
5 × 10^15^	60.50 ± 0.42	4.14 ± 0.16
1 × 10^16^	55.85 ± 0.30	4.08 ± 0.19
2 × 10^16^	53.78 ± 0.89	3.58 ± 0.31

The pristine sample exhibits a surface RMS roughness of 6.92 ± 0.22 nm. After implantation, the RMS roughness decreases to about 3.58 ± 0.31 nm at the highest fluence, indicating smoothening of the films. The particle size is found to decrease from 74.41 ± 0.71 nm (pristine) to 53.78 ± 0.89 nm (highest fluence). The decrease in particle size and RMS roughness can be ascribed to the rearrangement of surface atoms due to the elastic collisions. This leads to the evolution of small ZnO particles due to the breaking of clusters by the transfer of energy from incident ions. Kahng et al. [31] presented a nonlinear theory that explains the mechanism of the evolution of nanostructures on ion beam-implanted surfaces at normal incidence. According to this theory, in the early stages, sputtering leads to the formation of tiny wavy perturbations induced via instabilities created by the ion beam. These instabilities are followed by a surface relaxation process, which leads to the smoothening of the surface and is also mentioned as negative surface tension by others [32–33]. This process causes the breaking of larger structures into smaller ones. Thus, one can tune the surface morphology of films using an inert ion beam through a competition between surface diffusion and ion erosion processes [34–35].

#### Field-emission scanning electron microscopy

**Figure 8:**
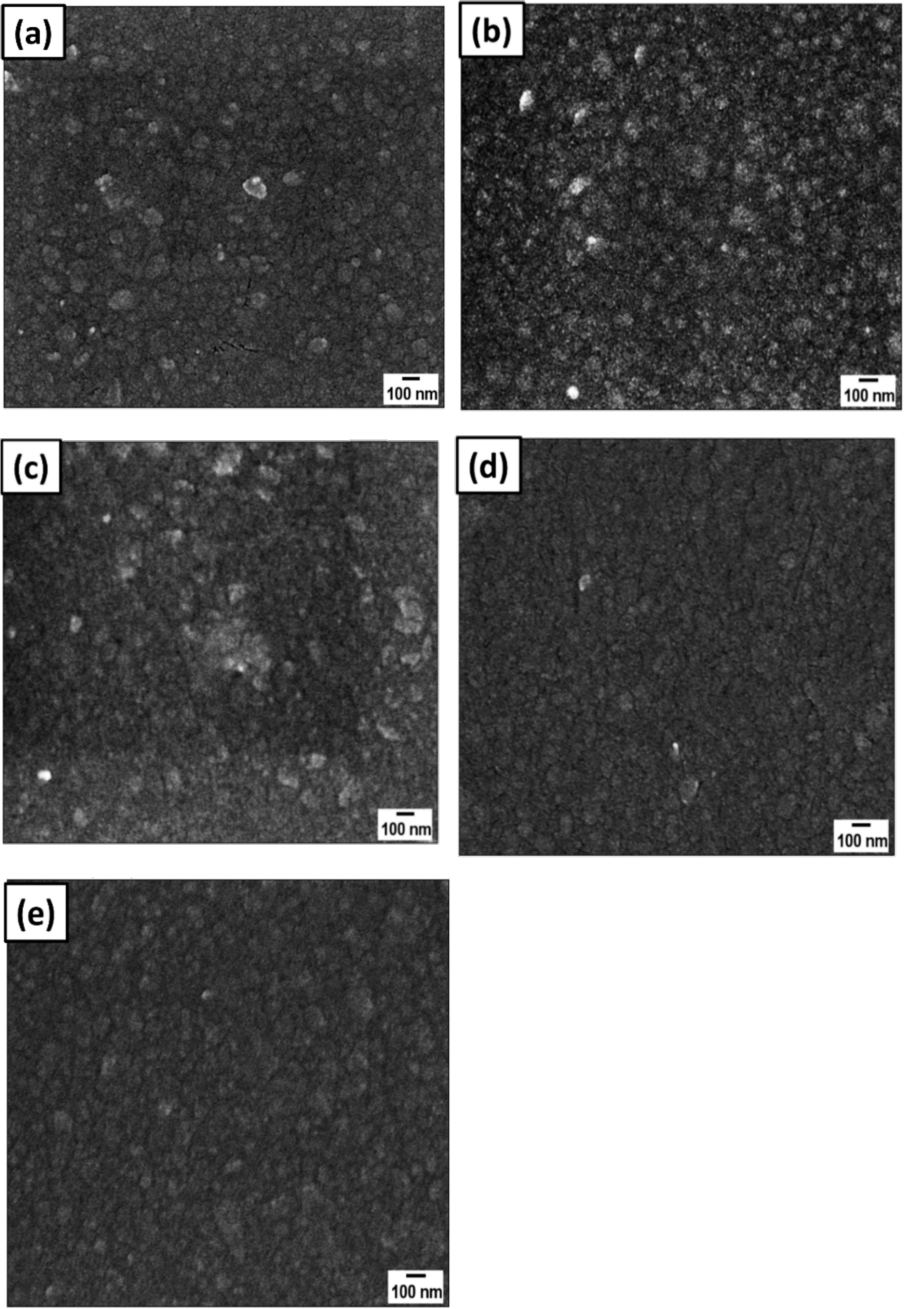
FESEM images of pristine (a) and Ar^+^-implanted ZnO films at fluences of (b) 1 × 10^15^, (c) 5 × 10^15^, (d) 1 × 10^16^, and (e) 2 × 10^16^ ions·cm^−2^.

The surface morphology of pristine and 30 keV argon-implanted ZnO films was also studied by FESEM. Figure 8 shows the FESEM images of pristine and implanted films. To deduce the change in surface RMS roughness and grain size of films after implantation, FESEM images have been processed with Image J software [36] and the results are given in Table 5.

**Table 5:** Variation in grain size and RMS roughness of pristine and implanted ZnO films.

Fluence (ions cm^−2^)	Average grain size (nm)	RMS roughness (nm)

pristine	63.24 ± 2.98	17.7 ± 0.43
1 × 10^15^	51.53 ± 1.58	16.8 ± 0.23
5 × 10^15^	43.80 ± 3.08	15.5 ± 0.58
1 × 10^16^	38.58 ± 0.67	12.6 ± 0.28
2 × 10^16^	25.22 ± 2.91	11.8 ± 0.68

It is observed from Figure 8 that average grain size and surface RMS roughness reduce with ion fluence. As the implantation dose of argon ions increases, the RMS roughness decreases from 17.8 ± 0.33 to 11.8 ± 0.68 because of inverse coarsening and fragmentation of nanostructures, leading to the smoothening of films. According to Paramanik et al. [33], surface smoothening can be associated with a decrease in the crystallinity of films. At high fluences, the density of electronic excitation increases, and covalent bonds in the lattice are weakened. This leads to relaxation, which causes surface smoothening. The stoichiometry of pristine and implanted samples evaluated using EDS analysis are shown in Table 6. Because of the native oxide layer on the Si substrate, the oxygen content contains contributions from both SiO_2_ and ZnO.

**Table 6:** EDS analysis of the pristine ZnO film and the film with the highest implanted dose.

Fluence (ions·cm^−2^)	O content (atom %)	Si content (atom %)	Zn content (atom %)	Ar content (atom %)

pristine	48.8	27.7	25.5	–
2 × 10^16^	47.2	28.7	23.8	0.3

The variations in grain size and RMS roughness of ZnO films with increase in ion fluence follow the same trend in AFM and FESEM analyses, but with different magnitudes. This is because of the greater sensitivity of AFM closer to the surface, while FESEM measures further inside the sample.

### Optical analysis

**Figure 9:**
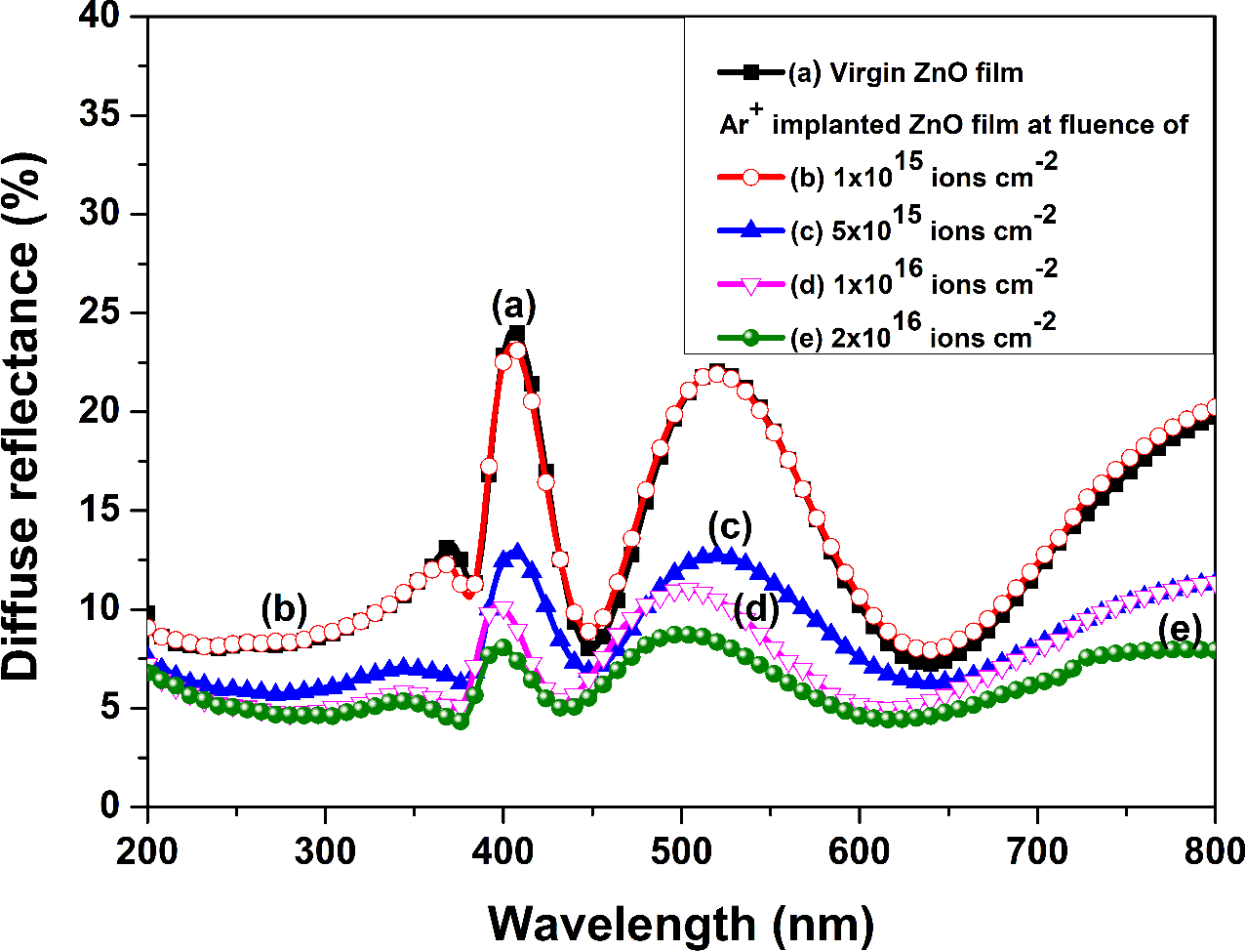
Diffuse reflectance spectra of (a) pristine and Ar^+^-implanted ZnO films at various fluences, viz. (b) 1 × 10^15^, (c) 5 × 10^15^, (d) 1 × 10^16^, and (e) 2 × 10^16^ ions·cm^−2^.

Figure 9 shows diffuse reflectance spectra of pristine and implanted ZnO films at different Ar^+^ ion fluences. The reflectance spectra of all the samples exhibit oscillating behavior, which can be attributed to interference phenomena due to differences in film refractive index and substrate refractive index.

This behavior of the spectra indicates the formation of smooth and uniform films on the quartz substrate [37]. The spectra of all samples show a sudden rise in reflectance above 370 nm, which represents the ZnO fundamental absorption edge. With the increase in argon ion fluence, the diffuse reflectance was found to decrease, which is related to the decrease in the surface RMS roughness also reported in AFM analysis. Thus, implanted ZnO films can be employed as an antireflection coating in optoelectronic devices [38].

The diffuse reflectance of the films can be used to calculate the associated Kubelka–Munk function, which is equivalent to the absorption spectra [39–40]. This paves the way to calculate the optical bandgap of the implanted films. The Kubelka–Munk function *F*(*R*) is determined employing diffuse reflectance by the following relation [41]:


[7] F(R)=(1−R)22R=αs.

**Figure 10:**
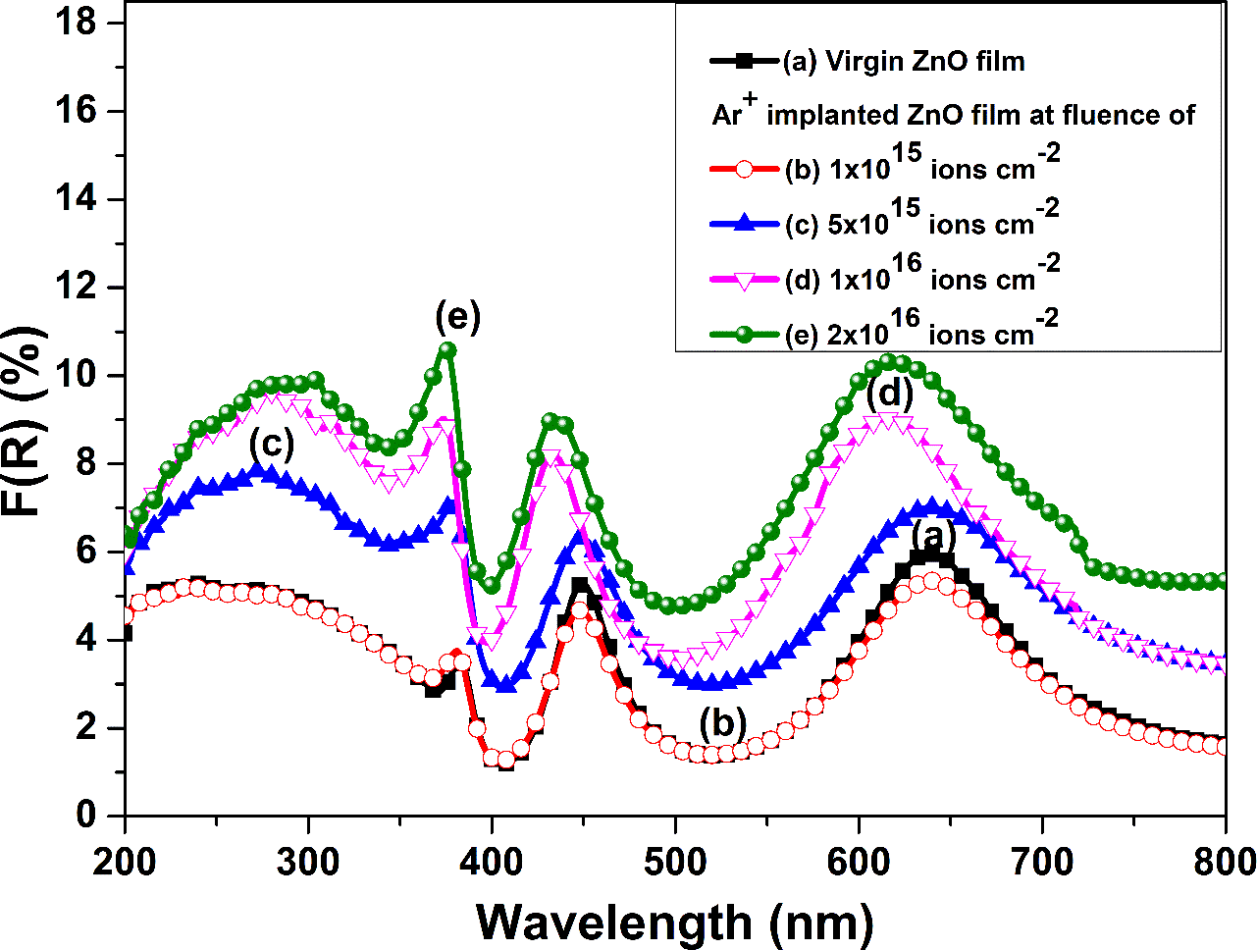
Kubelka–Munk function *F*(*R*) related to (a) pristine and Ar^+^-implanted ZnO films at various fluences, viz. (b) 1 × 10^15^, (c) 5 × 10^15^, (d) 1 × 10^16^, and (e) 2 × 10^16^ ions·cm^−2^.

Here *R* is the diffuse reflectance of the samples; *s* and α correspond to scattering and absorption coefficients, respectively. The scattering coefficient does not depend on the wavelength. Thus, *F*(*R*) becomes proportional to α. It has been observed that with the rise in ion fluence, *F*(*R*) of the films increases (Figure 10). This points towards the degradation of the crystal quality of ZnO films with disordering of atoms and defects in the films. This causes an increased absorption of UV and visible light. Moreover, additional peaks are observed, centered at around 450 and 650 nm, which are ascribed to the presence of defects like oxygen vacancies, oxygen interstitials, zinc vacancies, and zinc interstitials. The defects lead to the formation of sub-bandgap levels [42–43]. Further, the peak positions of these absorption peaks shift towards shorter wavelengths with an increase in ion fluence, which is coherent with a decrease in particle size. This depicts the effect of the surface morphology on the optical response of implanted films [44]. The intensity of these absorption peaks rises with the increase in ion fluence, which points towards an increase in defects as described in GXRD and Raman studies.

The optical bandgap (*E*_g_) values of samples have been estimated employing Tauc’s relation [45]:


[8] $${\left(\alpha h\nu \right)}^{n}=C\left(h\nu -E_{\text{g}}\right),$$

where α and *h*ν are absorption coefficient and photon energy, respectively, *C* represents constant, and *n* elucidates the transition type (*n* is 2/3 for forbidden direct, 2 for allowed direct, 1/3 for forbidden indirect, and 1/2 for allowed indirect transitions). The above equation has been evaluated regarding all possible *n* values. It is observed that for the present study, *n* = 2 holds good. Also, α is proportional to *F*(*R*), which modifies Equation 8 to:


[9] $${\left(F\left(R\right)\cdot h\nu \right)}^{2}\propto \left(h\nu -E_{\text{g}}\right).$$

**Figure 11:**
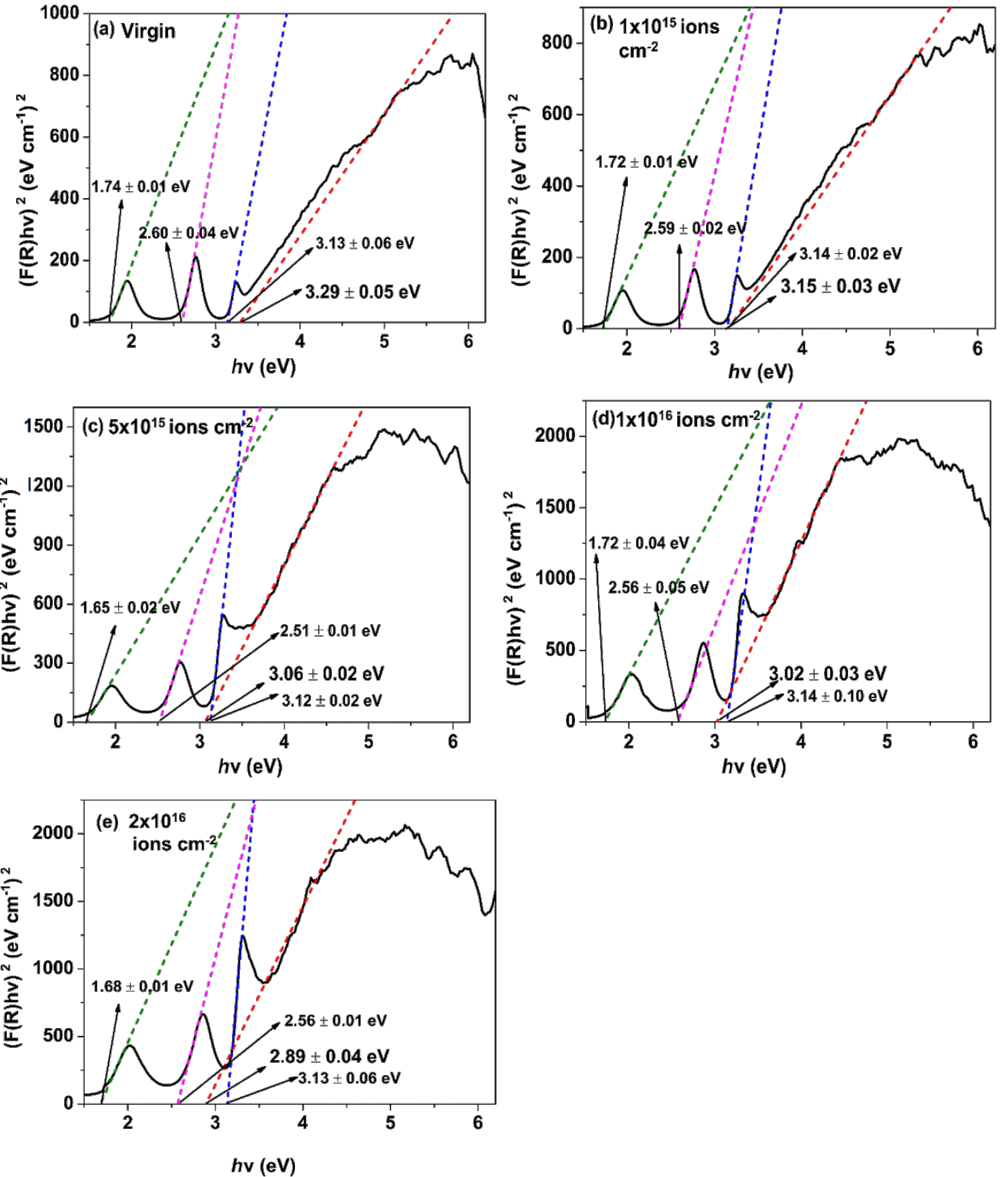
Tauc’s plot of (a) pristine and Ar^+^-implanted ZnO films at various fluences, viz. (b) 1 × 10^15^, (c) 5 × 10^15^, (d) 1 × 10^16^, and (e) 2 × 10^16^ ions·cm^−2^.

Extrapolation of the linear region of the (*h*ν·*F*(*R*))^2^ versus (*h*ν) plot to the energy axis is used to find optical bandgap values. Figure 11 depicts the different bandgap values, out of which the highest value of the bandgap values in each plot indicate the fundamental bandgap value, while the other three values represent sub-bandgap absorptions due to defects.

The optical bandgap values decrease after implantation from 3.29 ± 0.05 eV to 2.89 ± 0.04 eV with the rise in ion fluence. This is assigned to the emergence of defect-trapping levels between valence band and conduction band [46]. These trapping levels can be acceptor level or donor levels present at the top of the valence band or at the bottom of the conduction band, respectively. This results in a decrease in the energy separation between the valence band and the conduction band. Also, the sub-bandgap values decrease with increase in ion fluence as shown in Figure 11. Generally, implanted ions lead to the production of point defects, which act as trapping centers and affect the optical absorption [47].

Thus, a progressive decrement in bandgap values with increasing fluence is ascribed to lattice disorder due to argon ion implantation. Moreover, we have observed higher reductions of optical bandgap values than other earlier studies using low-energy ion beams [3,12]. Thus, low-energy argon ion implantation of ZnO films provides us with an approach to fabricate advanced materials having smoother surfaces, lower particle sizes, lower bandgap, and higher absorption in the UV region. This amplifies the data storage capacity and energy efficiency of ZnO films [13].

The implantation-induced structural disorder is reflected in terms of Urbach energy, which is defined as the band tail energy and can be calculated using the Urbach edge rule. Near the band edges, the absorption coefficient varies exponentially with photon energy [45]:


[10] $$\alpha \left(\lambda \right)=\alpha_{0}\exp\frac{h\nu }{E_{0}}.$$

**Figure 12:**
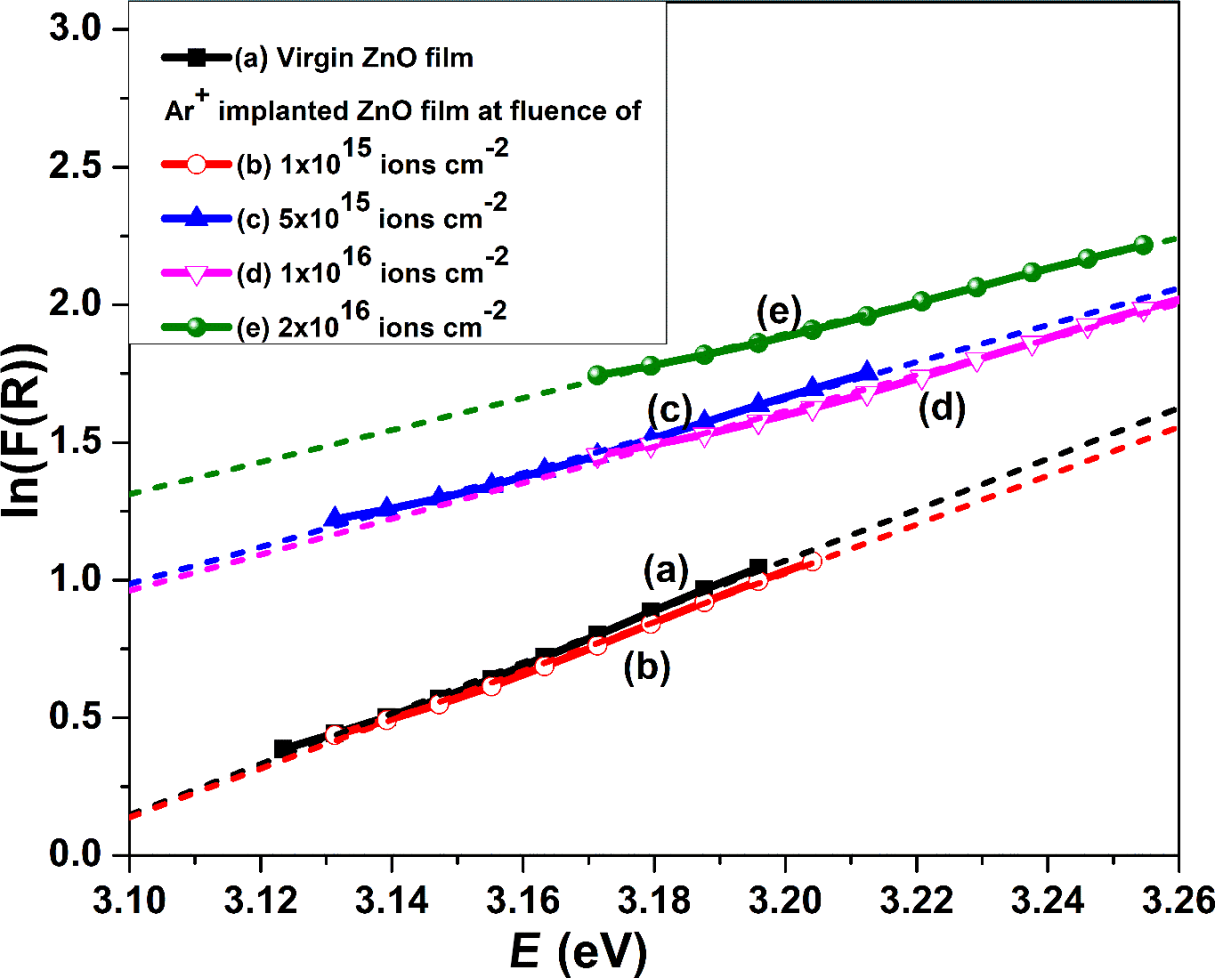
Plot of ln(*F*(*R*)) versus *E* for (a) pristine and Ar^+^-implanted ZnO films at various fluences, viz. (b) 1 × 10^15^, (c) 5 × 10^15^, (d) 1 × 10^16^, and (e) 2 × 10^16^ ions·cm^−2^.

Here, α_0_ represents a constant, α is the absorption coefficient, and *E*_0_ stands for the Urbach energy, which is calculated by taking the inverse of the slope of the plot between ln(α(λ)) and photon energy (*E* = *h*ν). Since α is proportional to *F*(*R*), the plot of ln(*F*(*R*)) versus *E* is employed to estimate Urbach energy. The dependence of ln(*F*(*R*)) on *E* for pristine and implanted ZnO films at various fluences is shown in Figure 12.

**Figure 13:**
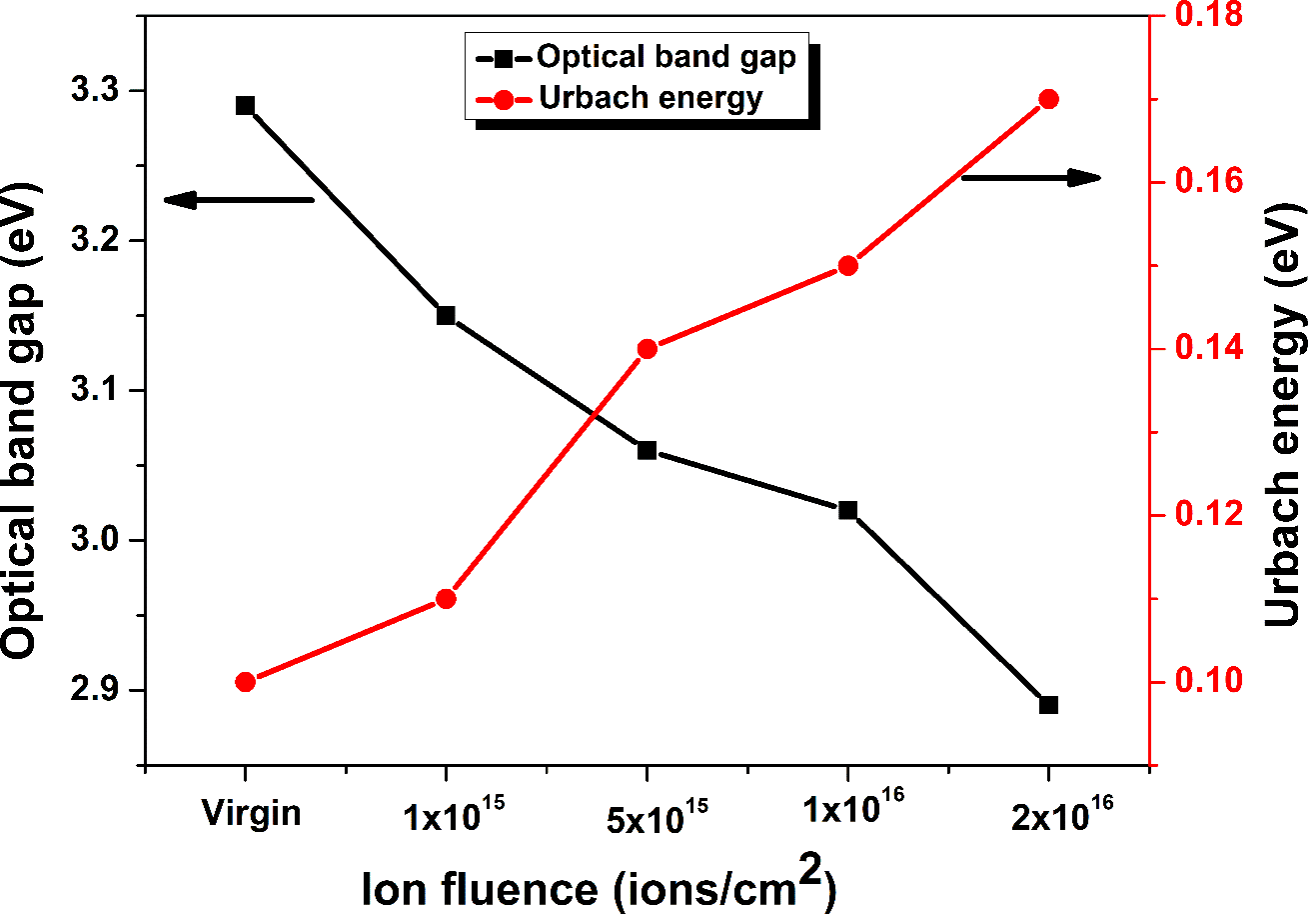
Variation in optical bandgap and Urbach energy values of pristine and 30 keV Ar^+^-implanted ZnO films at various fluences.

The value of Urbach energy for pristine and implanted ZnO films at fluences of 1 × 10^15^, 5 × 10^15^, 1 × 10^16^, and 2 × 10^16^ ions·cm^−2^ rises from 0.10 to 0.17 eV as shown in Figure 13. The increase in Urbach energy and decrease in optical bandgap (Figure 13) with the rise in argon ion fluence can be ascribed to implantation-induced structural disorder, which is coherent with GXRD and Raman analysis.

## Correlations

This anomalous behavior of Raman modes can be attributed to the fact that the incorporation of lattice defects and disorder by energetic ions leads to translational symmetry loss. This results in the breaking of the wave vector *k* = 0 selection rule required for Raman scattering from different parts of the Brillouin zone. Thus, scattering occurs from the whole Brillouin zone [48]. This can be correlated with the diminishing of the E_2_ (high) mode, enhancement in the disorder band (101–200 cm^−1^), and broadening of the symmetry-disallowed A_1_ (LO) Raman mode at the K–M point of the Brillouin zone at higher fluences. Moreover, AFM studies reveal grain size reduction leading to the enhancement in the density of grain boundaries. This generates an intrinsic electric field, which in turn evolves Raman optical modes [49]. Further, the fall in intensity of the E_2_ (high) mode is corroborated with GXRD studies, which revealed a decrease of the (002) peak and an increase in lattice strain along the *c* axis with increasing argon ion fluence. Besides this, the lattice defects induce distortion in the lattice, which leads to a decrease in the bandgap and an increase in Urbach energy due to the formation of bands that accumulate the defects and an increase in carrier concentration in the form of oxygen vacancies. Therefore, the evolution of different Raman modes and softening of 15 cm^−1^ of the A_1_ (LO) mode in implanted ZnO films can be ascribed to the phonon localization due to lattice defects, reduction in grain size, and structural strain.

## Conclusion

ZnO films have been investigated before and after Ar^+^ implantation to study the effect of ion fluence on various properties of the films. GXRD pattern reveals a decline in crystallinity along the *c* axis with the rise in ion fluence. Implanted ZnO films show the increase and decrease in intensity of A_1_ (LO) and E_2_ (high) Raman modes, respectively, with increasing argon ion fluence. The E_2_ (low) mode merges with a disorder-induced broad band at higher fluences. The peaks centered at 577 and 554 cm^−1^ in the deconvoluted spectrum of the A_1_ (LO) mode in implanted films are ascribed to oxygen vacancies and zinc interstitials, respectively. The film implanted at the highest fluence exhibits the smoothest surface and lowest grain size, which boosts light absorption and lower reflection. The optical bandgap values of ZnO films declined from 3.29 to 2.89 eV. Thus, we conclude that low-energy ion beams open a wide perspective for controlling the structural and optical characteristics of ZnO films, which makes them potential candidates for integrated optoelectronic devices.

## Data Availability

Data generated and analyzed during this study is available from the corresponding author upon reasonable request.
